# Economic Evaluations of Algorithm-Enabled Remote Monitoring of Cardiac Implantable Electronic Devices: Systematic Review

**DOI:** 10.2196/106939

**Published:** 2026-11-13

**Authors:** Hanan Daghash, Rohan Pandey, Ryan P W Kenny, Cristina Fernandez-Garcia, Edward Meinert, Gurdeep S Sagoo

**Affiliations:** 1 Population Health Sciences Institute, Newcastle University, Newcastle upon Tyne, England, United Kingdom; 2 Translational and Clinical Research Institute, Newcastle University, Newcastle upon Tyne, England, United Kingdom; 3 Department of Primary Care and Public Health, School of Public Health, Imperial College London, W6 8RP, London, United Kingdom, 33 7824446808

**Keywords:** systematic review, economic evaluation, cost-effectiveness, cardiac implantable electronic devices, CIEDs, remote monitoring, heart failure

## Abstract

**Background:**

Algorithm-enabled remote monitoring using cardiac implantable electronic devices (CIEDs) is increasingly used to support earlier detection of clinical deterioration in heart failure and in at-risk populations. However, the economic value of these technologies remains uncertain because intervention design, implementation, and evaluation methods vary.

**Objective:**

This study aimed to systematically identify, appraise, and synthesize full economic evaluations of algorithm-enabled CIED remote monitoring in adults with established heart failure or at risk of heart failure.

**Methods:**

A systematic review was conducted in accordance with the PRISMA (Preferred Reporting Items for Systematic Reviews and Meta-Analyses) 2020 guidelines. Searches were performed in MEDLINE, Embase, Scopus, Web of Science, and the Cochrane Library, with supplementary searches of health technology assessment sources. Eligible studies reported full economic evaluations of algorithm-enabled remote monitoring linked to CIEDs. Data were extracted using a structured form and synthesized narratively due to heterogeneity. Methodological and reporting quality were assessed using the CHEQUE (Criteria for Health Economic Quality Evaluation) checklist and CHEERS (Consolidated Health Economic Evaluation Reporting Standards) 2022 statement. One reviewer screened records and extracted the data. A second reviewer independently checked subsets of the screening decisions and extracted the data.

**Results:**

Ten economic evaluations were included. They used models, trial data, or observational data; some were reported within health technology assessments. Substantial heterogeneity was observed in population definitions, intervention characteristics, algorithm types, monitored parameters, clinical response pathways, and economic methods. Cost-effectiveness and cost-utility analyses were most common, with time horizons ranging from short-term follow-up to lifetime modeling. Several studies reported economically favorable findings, including dominance (ie, less costly with better health outcomes) or cost-effectiveness. However, outcomes varied across studies, and favorable estimates often relied on assumptions about reduced hospitalization and length of stay, while quality-adjusted life year gains were generally modest. Reporting of key implementation factors, including alert burden and diagnostic performance, was limited.

**Conclusions:**

There is economic evidence for algorithm-enabled CIED remote monitoring suggesting potential value in some settings, but the evidence remains methodologically heterogeneous and difficult to compare directly across studies. Findings are sensitive to differences in intervention design, modeling approaches, and clinical input data, which limit certainty and transferability. More robust and standardized evidence is needed before economic conclusions can be applied confidently across different devices, algorithms, and health care settings.

## Introduction

Heart failure (HF) is a chronic, progressive syndrome associated with substantial morbidity, mortality, and health care use [1]. Despite therapeutic advances, outcomes following HF admission remain poor, with the UK National Heart Failure Audit reporting 39% all-cause mortality at 1 year after discharge and 9% in-hospital mortality [2]. Because HF is characterized by periods of relative stability interrupted by episodes of worsening or decompensation [3-5], care models have increasingly shifted toward proactive surveillance and earlier detection of deterioration [6]. Remote monitoring via cardiac implantable electronic devices (CIEDs), including pacemakers, implantable cardioverter-defibrillators (ICDs), cardiac resynchronization therapy pacemakers (CRT-Ps), and cardiac resynchronization therapy defibrillators (CRT-Ds), is increasingly used to enable earlier identification of clinically important changes [1]. CIEDs record information about device performance and the patient’s health. A home monitor or compatible phone sends these data to a secure platform. Data may be sent at set times or after the device detects an event [7]. Some systems use predefined rules to generate an alert when a condition is met. Others combine several device measurements to estimate the patient’s risk of worsening HF [8]. A clinician can review the alert and contact the patient or change their care if needed [9]. The same monitoring program may use scheduled transmissions, event-driven transmissions, and automated alerts. This review, therefore, focused on algorithm-enabled systems as these features may influence both clinical pathways and the structure of economic evaluation.

The economic evaluation of algorithm-enabled remote monitoring may pose additional challenges compared with evaluating medicines and medical devices without remote monitoring functions. Digital health interventions are often interactive, rapidly evolving, and dependent on user engagement. At the same time, narrow health service perspectives and cost per quality-adjusted life year (QALY) metrics alone may not fully capture the broader societal consequences and service-level impacts relevant to digital health interventions [10]. In addition, value may depend on diagnostic performance, the deployment model, referral thresholds, the consequences of false positives and false negatives, and supporting digital infrastructure [11]. These issues may be relevant in algorithm-enabled pathways, where alert burden, clinical workflow, and downstream resource use may influence cost-effectiveness estimates. At the system level, many health technology assessment (HTA) frameworks have been developed for pharmaceuticals and medical devices without remote monitoring functions. They may not adequately capture the iterative, infrastructure-dependent, and context-sensitive nature of digital technologies [12]. The challenge is whether economic evaluations of these systems capture patient and caregiver time, changes in clinical workload, and digital infrastructure costs alongside health care costs and QALYs.

Evidence on the clinical, service, and economic impact of CIED remote monitoring remains mixed and depends on the monitoring system, patient population, and care pathway. Some studies have reported limited effects on major outcomes such as all-cause mortality or cardiovascular hospitalization despite improvements in process outcomes such as faster clinical decision-making and fewer scheduled visits [13]. Others have reported reductions in HF hospitalizations, shorter lengths of stay, lower mortality risk, and lower overall health care service use [14,15], with better outcomes among patients with higher monitoring adherence [16]. Economic evaluations have generally applied cost-effectiveness and cost-utility frameworks, and many have reported economically favorable findings. There has been trial-based evidence suggesting modest QALY gains and lower patient-borne costs [17]. Model-based evaluations, particularly those using longer time horizons, have reported cost-effectiveness or cost-saving conclusions [18-21]. However, findings remain sensitive to alert frequency, algorithm design, device type, clinical response pathways, reimbursement assumptions, analytic perspective, costing methods, and model structure [22,23], limiting comparability and transferability.

Most reviews have focused primarily on clinical outcomes and reported mixed effects without a detailed economic appraisal [22,24]. Raes et al [25] examined CIED telemonitoring across broader indications and did not specifically address algorithm-enabled systems in HF populations. The National Institute for Health and Care Research (NIHR) HTA report by Kenny et al [26] systematically reviewed clinical and economic evidence for 4 HF monitoring algorithms and developed an independent economic model. Consequently, a gap remains in understanding how algorithm-enabled remote monitoring has been evaluated economically, how methodological choices may shape the findings, and what limitations hinder comparability and policy interpretation.

Taken together, the clinical and economic value of algorithm-enabled CIED remote monitoring remains unclear. The available evidence is difficult to interpret because interventions, implementation contexts, and evaluation methods vary across studies. A systematic review is therefore needed to synthesize the existing evidence, examine the methodological approaches used, and identify the limitations that affect comparability and policy interpretation.

This systematic review aimed to identify, appraise, and synthesize full economic evaluations of algorithm-enabled remote monitoring using CIEDs to (1) describe the characteristics and methodological features of the included economic evaluations, including economic modeling structure, analytical perspective, and uncertainty analysis; (2) summarize the clinical outcomes, economic outcomes, and main cost-effectiveness findings reported across studies; and (3) identify methodological limitations and evidence gaps that may affect model development, comparability, and policy interpretation.

## Methods

### Reporting and Registration

This review has been reported in accordance with the PRISMA (Preferred Reporting Items for Systematic Reviews and Meta-Analyses) 2020 guidelines [27], and the protocol was developed in line with the PRISMA-P (Preferred Reporting Items for Systematic Reviews and Meta-Analyses Protocols) 2015 guidelines [28]. The protocol was prospectively registered in PROSPERO (CRD420251179324) and is publicly available in *JMIR Research Protocols* [29].

### Eligibility Criteria

Eligibility criteria were predefined using the population, intervention, comparator, outcome, and study design framework and are summarized in Table 1. Studies were eligible if they reported full economic evaluations of algorithm-enabled remote monitoring linked to CIEDs in adult HF or at-risk populations.

**Table 1.** Eligibility criteria based on the PICOS (population, intervention, comparator, outcome, and study design) framework.

Domain	Eligibility criteria
Population	Adults aged ≥18 years with heart failure, or populations defined in the primary studies as being at risk of heart failure, managed using cardiac implantable electronic devices (CIEDs^a^), including ICDs^b^, CRT-Ps^c^, CRT-Ds^d^, or pacemakers.
Intervention	Algorithm-enabled remote monitoring integrated with CIEDs to detect worsening heart failure or events and alert generation.
Comparator	Standard in-person follow-up, usual care without remote monitoring, or alternative remote monitoring strategies.
Outcomes	At least one economic outcome, including ICERs^e^, incremental costs, QALYs^f^, life-years gained, net monetary benefit, or related cost-effectiveness endpoints. Clinical outcomes and alert performance measures were also extracted where reported.
Study design	Full economic evaluations, including trial-based and model-based cost-effectiveness, cost-utility, and cost-benefit analyses. Cost-consequence analyses were also retained where relevant. Conference abstracts were included only when sufficient methodological and outcome data were available.
Exclusion criteria	Non-English studies; studies not evaluating device-linked remote algorithms; studies not reporting economic outcomes; protocols; trial registry entries without results; narrative reviews; and perspective papers.

a CIED: cardiac implantable electronic device.

b ICD: implantable cardioverter-defibrillator.

c CRT-P: cardiac resynchronisation therapy pacemaker*.*

d CRT-D: cardiac resynchronisation therapy defibrillator*.*

e ICER: incremental cost-effectiveness ratio*.*

f QALY: quality-adjusted life-year*.*

An eligible intervention used CIED remote monitoring with continuous connectivity or intermittent data transmissions. It generated an alert for clinical review when the data indicated possible worsening HF or an event. A commercial algorithm name was not required.

### Information Sources and Search Strategy

A systematic search was conducted between November and December 2025 in MEDLINE (Ovid), Embase (Ovid), Scopus, Web of Science, and the Cochrane Library. Supplementary searches were undertaken in the National Health Service Economic Evaluation Database, the National Institute for Health and Care Excellence, the Canadian Agency for Drugs and Technologies in Health, the international HTA database, and the National Institute for Health and Care Research Journals Library. The search strategy was developed in consultation with a medical librarian and combined controlled vocabulary and free-text terms related to CIEDs, remote monitoring, HF algorithms, and economic evaluation. The full search strategy is provided in Multimedia Appendix 1 [15,17-21,26,30-32].

### Data Management and Study Selection

All records were exported to EndNote (version 21; Clarivate Analytics) for duplicate removal and then imported into Rayyan (Rayyan Systems Inc) [33] for screening. Study selection was undertaken in 2 stages: title and abstract screening followed by full-text screening. One reviewer screened all records against the prespecified eligibility criteria. A second reviewer independently checked the first 476 titles and abstracts and the first 27 full-text reports made available for verification in Rayyan. Reviewers’ decisions remained hidden until both had completed their assessments. Any disagreements were resolved through discussion. A third reviewer was available but was not required. For full-text exclusions, one primary reason was recorded. Partial verification was used because of time and resource constraints. The study selection process was summarized using a PRISMA 2020 flow diagram.

### Data Extraction

Data were extracted using a structured Microsoft Excel–based form, tested on an initial sample of included studies, and refined iteratively through discussion with the review team before full data extraction. A second reviewer checked the extracted data against the source documents for 3 reports. The review team selected these reports without a formal sampling procedure. The 2 reviewers resolved any differences through discussion. A third reviewer was available but was not needed. Partial verification was used because of time and resource constraints.

### Quality Appraisal and Reporting Assessment

The methods and reporting of the included economic evaluations were appraised using the CHEQUE (Criteria for Health Economic Quality Evaluation) tool [34]. Reporting completeness was also assessed at the item level using the CHEERS (Consolidated Health Economic Evaluation Reporting Standards) 2022 statement [35].

This approach differed from the registered protocol, which proposed several appraisal tools based on study design. A single tool was used to support consistent descriptive appraisal across the different types of economic evaluation included in the review.

The first author (HD) completed both assessments. CHEQUE items were rated as “yes,” “somewhat,” “no,” or “not applicable.” “Yes,” “somewhat,” and “no” received scores of 1, 0.5, and 0, respectively. Methods and reporting percentages were calculated separately by dividing the sum of item scores by the number of applicable items and multiplying by 100. Not applicable items were excluded, and applicable items were weighted equally. CHEERS was used to assess reporting completeness. It was not used to assess methodological quality or risk of bias.

### Data Synthesis

Data were synthesized narratively because of substantial heterogeneity in study design, intervention type, algorithm specification, analytical perspective, model structure, time horizon, and reported outcomes. Structured summary tables and descriptive comparisons were used to synthesize study characteristics, intervention and alert workflow features, economic evaluation characteristics, model structure, clinical outcomes, economic outcomes, and main cost-effectiveness findings. No quantitative meta-analysis was undertaken due to substantial heterogeneity in design, setting, outcome measurement, perspective, and modeling assumptions.

## Results

### Study Selection

Figure 1 summarizes the study selection process. The database searches identified 3108 records. In the full-text assessment, 132 full texts were screened for eligibility. Of these, 122 (92.4%) were excluded. The review included 10 (7.6%) reports: 8 (80%) primary study reports and 2 (20%) HTA reports. The broad search retrieved records on remote care and economic outcomes beyond the scope of this review. Some broad search terms returned large numbers of records before the search sets were combined (Multimedia Appendix 1 [15,17-21,26,30-32]).

**Figure 1.**
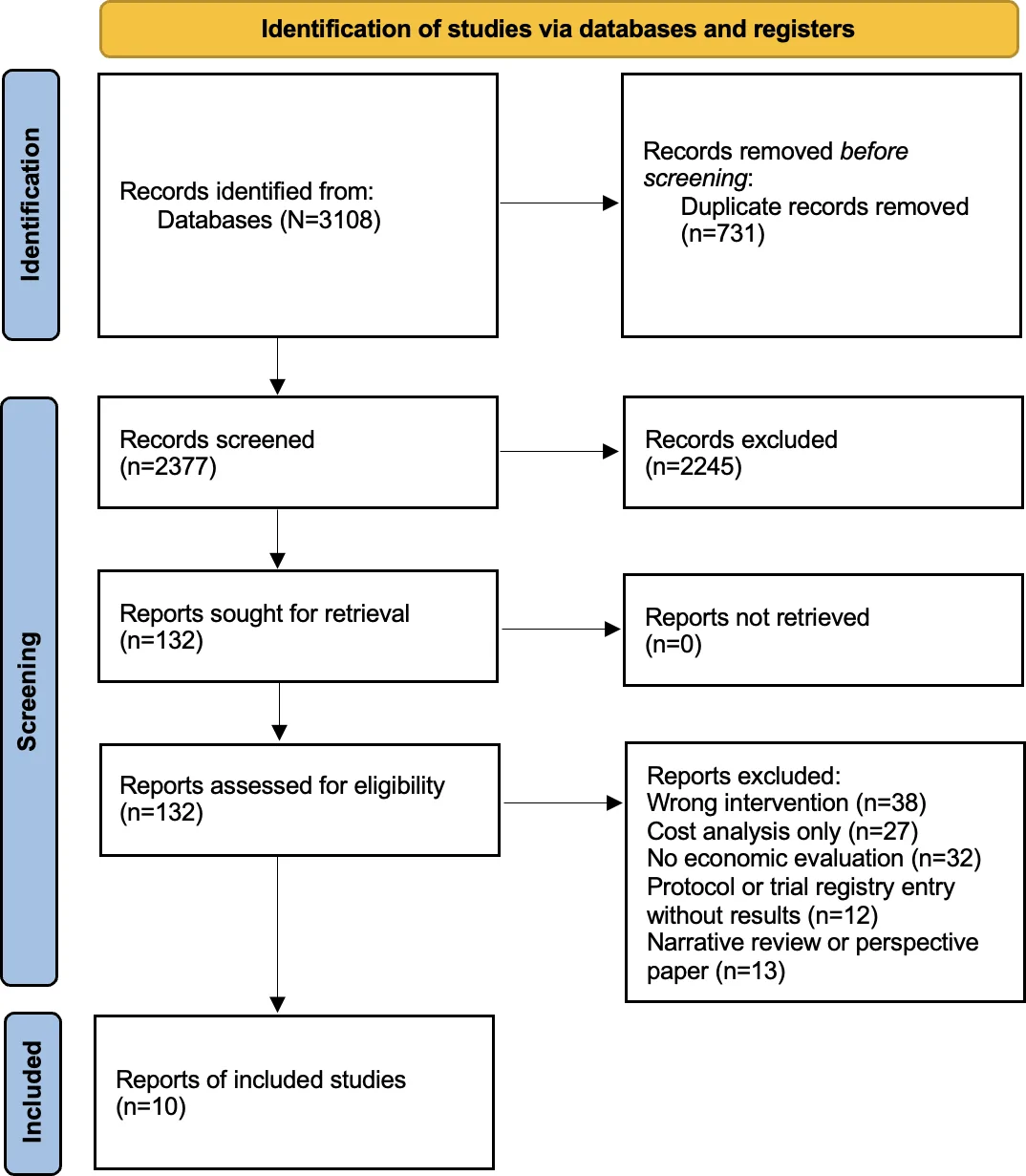
PRISMA (Preferred Reporting Items for Systematic Reviews and Meta-Analyses) flow diagram of the study selection process.

### Characteristics of Included Studies

Table 2 shows the 10 studies. Most were set in Europe or North America. Study designs included model-based economic evaluations, randomized controlled trials, prospective nonrandomized clinical studies, population-based cohort studies, real-world multicenter comparative studies, systematic reviews, meta-analyses, and HTAs.

**Table 2.** Characteristics of included studies.

Study (year)	Country	Economic evaluation design	Main clinical evidence source	Intervention	Comparator	Population	Funding source
Sequeira et al (2020) [21]	France	Model-based cost-utility analysis published within a systematic review	ECOST^a^ clinical evidence; EVOLVO^b^ utility values	Not reported at the study level; constituent RCTs^c^ included OptiVol-enabled devices (Medtronic) with OptiVol alert activation or deactivation settings	Conventional follow-up	Adults ≥18 years with ICD^d^ ± CRT-D^e^; 17 RCTs (n=10,229); mean age 54.7-69.5 years; 72%-88% male; mean LVEF^f^ 20%‐40%	No external funding
Health Quality Ontario (2018) [31]	Canada (Ontario)	Model-based economic evaluations published within an HTA^g^	Evidence synthesis and published model inputs; individual trial (EVOLVO-TRUST) contributions to each model were not fully established	RM^h^ plus clinic visits	Clinic visits alone	Model 1: adults with HF^i^ and ICD/CRT-D (mean age 65 years; 70% male; NYHA^j^II); model 2: adults with arrhythmia and pacemakers (mean age 70 years; 65% male); maintenance phase (≥3 months after implant)	Ontario Health
Zanaboni et al (2013) [17]	Italy	Cost-utility analysis alongside an RCT	EVOLVO trial	RM via Medtronic CareLink Home Monitor	Conventional in-person follow-up	200 patients with HF with ICD/CRT-D (RM: n=99; SC^k^: n=101)	Italian Ministry of Health; Regione Lombardia; Medtronic Italia (technical support only)
Hummel et al (2019) [18]	United States	Model-based economic evaluation	PREDICT RM observational cohort	Remote patient monitoring	Conventional in-clinic follow-up	15,254 ICD recipients aged 65‐89 years (Medicare); HF present in 59.8%	Boston Scientific Corporation
Burri et al (2013) [19]	United Kingdom	Model-based cost-consequence analysis	Published trial evidence, including TRUST, and additional model inputs	Daily continuous RM (BIOTRONIK Home Monitoring)	Conventional in-clinic follow-up	ICD/CRT-D recipients undergoing primary or secondary prevention; mean age 65 years at implant; ICD 62.8% and CRT-D 37.2%. Model based on pooled RCT evidence weighted to the UK device mix; included HF-related events	Biotronik
Kenny et al (2025) [26]	United Kingdom	Original model-based economic evaluations published within an HTA	Technology-specific evidence from the HTA review and company submissions.	Algorithm-based RM of HF risk (CorVue, HeartInsight, HeartLogic, and TriageHF Plus)	No algorithm-based RM	Adults >18 years with CIEDs^l^; established chronic HF or at-risk of new-onset HF; includes CRT-P^m^, CRT-D, ICD, and pacemaker recipients; subgroups by NYHA class and prior HF hospitalization	NIHR^n^ Evidence Synthesis Programme (NIHR135894)
de Graaf et al (2024) [32]	Netherlands, Germany, and France	Economic evaluation alongside an RCT	REMOTE-CIED trial	RPM using LATITUDE system; annual outpatient visit plus remote checks more than every 6 months	In-clinic monitoring (usual care) with outpatient visits every 3‐6 months	595 first-time ICD recipients with symptomatic HF (LVEF ≤35%; NYHA II-III); age 18‐85 years	Boston Scientific Corporation
Chew et al (2022) [15]	Canada (Alberta)	Model-based economic evaluation informed by an observational cohort	Alberta cohort of patients with de novo implanted ICDs or CRT-Ds	RM program enrollment after initial in-person clinic visits (within the first year after implant)	In-clinic follow-up alone	2799 adults ≥18 years with de novo ICD or CRT-D; median age 66.5 years; 22.3% female; 15% CRT-D; HF comorbidity in 44.3% of RM group; median follow-up 50.3 months	Alberta Innovates Health Solutions; government of Alberta
Ricci et al (2017) [20]	Italy	Economic evaluation alongside a prospective observational study	TARIFF study	RM (Merlin@home; alert-based continuous monitoring) with in-person visits at enrollment and 12 months; remote follow-up at 3, 6, and 9 mo	Standard care with in-person follow-up at 3, 6, 9, and 12 months	209 CIED recipients (SC: n=107; RM: n=102); mean age approximately 69 years; approximately 85% male; mean LVEF approximately 32% ; NYHA III-IV: 29%-38%; predominantly ischemic cardiomyopathy; primary prevention 78%	St. Jude Medical Italy
Ahmed et al (2026) [30]	United Kingdom	Model-based economic evaluation informed by an observational study	TriageHF Plus clinical program	TriageHF Plus (device-based RM pathway triggered by HF alerts from CIEDs)	Usual care: guideline-based HF management and standard CIED follow-up without action on HF alerts	758 TriageHF-compatible CIED recipients (TriageHF Plus: n=443; usual care: n=315); mean age 67.8 years; 27.7% female; majority with established HF	Medtronic Inc

a ECOST: Effectiveness and Cost of ICD Follow-up Schedule With Telecardiology.

b EVOLVO: Evolution of Management Strategies of Heart Failure Patients With Implantable Defibrillators.

c RCT: randomized controlled trial*.*

d ICD: implantable cardioverter-defibrillator.

e CRT-D: cardiac resynchronisation therapy defibrillator.

f LVEF: left ventricular ejection fraction.

g HTA: health technology assessment.

h RM: remote monitoring.

i HF: heart failure.

j NYHA: New York Heart Association.

k SC: standard care.

l CIED: cardiac implantable electronic device.

m CRT-P: cardiac resynchronisation therapy pacemaker.

n NIHR: National Institute for Health Research.

Studies reported sample characteristics in varying ways (Table 2). Sample sizes ranged from 200 participants to 15,254 [17,18], while another study synthesized evidence from 17 randomized controlled trials comprising 10,229 participants [21]. Where reported, study populations generally consisted of older adults, and several studies included predominantly male participants [15,19,20,30,31]. Some studies focused specifically on patients with established HF [17,32], whereas others included broader CIED populations with either mixed HF status or HF risk [19,26,30]. Baseline clinical characteristics and comorbidities were not consistently described across studies. HF prevalence was explicitly reported in 2 studies [15,18]. In contrast, other studies primarily described HF severity indicators such as New York Heart Association (NYHA) class or left ventricular ejection fraction [20,21,31,32] or eligibility based on monitoring pathway and implant status [19,26] rather than detailed comorbidity profiles. Funding sources were also mixed (Table 2).

The following are the main clinical evidence sources identified from the included evaluations. Zanaboni et al [17] evaluated the Evolution of Management Strategies of Heart Failure Patients With Implantable Defibrillators (EVOLVO) trial [36]. Sequeira et al [21] used EVOLVO utility values [36] and evidence from Effectiveness and Cost of ICD Follow-up Schedule With Telecardiology (ECOST) [37]. Health Quality Ontario [31] also used EVOLVO data [36]. EVOLVO was therefore a shared clinical evidence source across these 3 economic evaluations.

### Intervention, Algorithm, and Alert Workflow Characteristics

The included studies showed substantial heterogeneity in remote monitoring system specifications, algorithm types, monitored parameters, and clinical response pathways (Table 3).

**Table 3.** Characteristics of remote monitoring systems and clinical response pathways.

Study (year)	Reported system or algorithm (manufacturer)	Algorithm type	Monitored parameters	Clinical response pathway
Sequeira et al (2020) [21]	Not reported	Not reported	Clinical parameters (eg, arrhythmia)	Transmit data at scheduled times or upon detection event and generate alerts and providing information to clinicians.
Health Quality Ontario (2018) [31]	CardioMessenger (Biotronik) CareLink (Medtronic) LATITUDE (Boston Scientific) Merlin.net (Abbott) SmartView (LivaNova)	Rule-based alert system	Device function and integrity Battery status and depletion Lead performance and failure Arrhythmia detection (including inappropriate shocks) ICD^a^ therapy delivery Physiological parameters	Remote clinician review of transmitted data; detection of device-related and clinical problems prompts early intervention, including medication adjustment or unscheduled in-person follow-up
Zanaboni et al (2013) [17]	CareLink Network Medtronic	Rule-based alert system	Device function parameters HF^b^-related parameters (eg, intrathoracic impedance for fluid accumulation monitoring) Atrial arrhythmias ICD therapy delivery	Clinician web-based review of transmitted data; unscheduled remote follow-up triggered by wireless alerts; telephone contact with patient if clinically indicated
Hummel et al (2019) [18]	PREDICT RM database: the Boston Scientific Corporation ALTITUDE database	Not reported	Device-related parameters Clinical parameters (eg, ventricular tachycardia and ventricular fibrillation)	Remote transmissions with follow-up visit
Burri et al (2013) [19]	BIOTRONIK Home Monitoring CardioMessenger II Biotronik	Rule-based alert system	Clinical parameters (eg, atrial fibrillation burden) Technical parameters (eg, abnormal lead impedance) Device function data	Early detection and remote review of clinically or technically relevant alerts; remote follow-up of home monitoring alerts between scheduled visits; in-person assessment if clinically required
Kenny et al (2025) [26]	CorVue + Merlin.net PCN (Abbott) HeartInsight + BIOTRONIK Home Monitoring (Biotronik) HeartLogic + LATITUDE NXT (Boston Scientific) TriageHF Plus + CareLink (Medtronic)	Algorithmic risk stratification (multi-parametric composite indices)	CorVue: ITI^c^ HeartInsight: composite score derived from 7 parameters (atrial burden, heart rate, and 5 additional parameters) HeartLogic: composite index derived from multiple implant-measured parameters TriageHF Plus: AT^d^/AF^e^ burden, ventricular rate during AT/AF, OptiVol fluid index, and additional physiological parameters	HF risk alert transmitted to HCPs^f^; stored device data reviewed; structured clinical management pathway including follow-up consultations and treatment adjustments as indicated
de Graaf et al (2024) [32]	LATITUDE Patient Management System Boston Scientific	Not reported	Predefined alert ICD device status	Transmitted data reviewed and acted upon by nurses, cardiologists, and/or ICD technicians according to each center’s established clinical protocol
Chew et al (2022) [15]	Not reported (population-based administrative data study; specific commercial RM systems not identified)	Not reported	Earlier detection of device malfunction or Changes in clinical status (eg, worsening heart failure and arrhythmias)	Daily communication between the CIED^g^ and home monitor; transmissions to the clinic were intermittent and event driven
Ricci et al (2017) [20]	Merlin@home St. Jude Medical (Abbott)	Rule-based alert system	Technical malfunctions Atrial arrhythmias Ventricular arrhythmias HF-related events ST segment changes	The majority of alerts were managed remotely, without the requirement for in-hospital attendance
Ahmed et al (2026) [30]	TriageHF Plus CareLink (Medtronic)	Algorithmic risk stratification (multi-parametric)	Cardiac rhythm Intrathoracic fluid levels Physical activity levels Daytime and nighttime heart rate Device-delivered therapies Irregular heartbeats Composite multi-parametric algorithm calculates patient’s 30-day HF event risk (low, medium, or high)	High-risk HF alerts reviewed by the HF team (typically an HF nurse); twice-weekly review of the RM^h^ platform; structured telephone assessment following alert; clinical management as indicated, including lifestyle advice and/or medication adjustment

a ICD: implantable cardioverter-defibrillator.

b HF: heart failure.

c ITI: intrathoracic impedance.

d AT: atrial tachycardia.

e AF: atrioventricular.

f HCP: healthcare professional.

g CIED: cardiac implantable electronic device.

h RM: remote monitoring.

#### Remote Monitoring Platforms, Systems, and Algorithms

The systematic review included different remote monitoring platforms and alert tools. Table 3 shows the reported names and manufacturers where available.

#### Algorithm Type

Algorithm type was classified according to the reported function of the system rather than the platform name. Several systems were described as rule-based alert systems (n=4) [17,19,20,31]. Two studies evaluated algorithmic risk stratification approaches based on multiparametric composite indexes for identifying increased risk of HF deterioration [26,30]. Algorithm type was not reported in 4 studies [15,18,21,32].

#### Monitored Parameters

Reported monitored parameters varied considerably across studies. Rule-based systems commonly monitored device function; technical integrity; and alert-based events, including battery status, lead performance, arrhythmias, and device therapy delivery [17,19,20,31]. Some studies also included HF-related or physiological parameters, such as intrathoracic impedance, atrial fibrillation burden, and other device-derived indicators of clinical deterioration [15,18,21,32]. In the studies by Kenny et al [26] and Ahmed et al [30], monitored inputs were multiparametric and used to generate composite HF risk estimates.

#### Alert Workflow and Clinical Response

Clinical response pathways ranged from structured clinician-led review to limited or absent reporting. Health Quality Ontario [31] described remote clinician review of transmitted data, with early intervention including medication adjustment or unscheduled in-person follow-up when clinically indicated. Burri et al [19] reported remote review of clinically or technically relevant alerts between scheduled visits, with in-person assessment when required. Zanaboni et al [17] described a clinician web-based review of transmitted data, with unscheduled remote follow-up and telephone contact if clinically indicated. In contrast, Ricci et al [20] reported that most alerts were managed remotely without hospital attendance. In the studies by Kenny et al [26] and Ahmed et al [30], high-risk HF alerts triggered structured review and follow-up processes, including health care professional assessment and clinical management, as indicated. de Graaf et al [32] reported that transmitted data were reviewed and acted upon in accordance with local center protocols. Other studies described clinical response pathways insufficiently [15,18,21].

### Economic Evaluation Characteristics

Table 4 summarizes the economic evaluation characteristics of the included studies, including economic analysis type, analytical perspective, time horizon, discounting, currency, price year, and sensitivity or uncertainty analysis.

**Table 4.** Economic evaluation characteristics of included studies.

Study (year)	Economic analysis type	Perspective	Time horizon	Discount rate	Currency	Price year	Sensitivity analysis
Sequeira et al (2020) [21]	Cost-utility analysis	French health care system	5 years	4% per annum (costs and outcomes; equivalent to 0.33% per month)	Euro	2017‐2018	Probabilistic sensitivity analysis (Monte Carlo simulation; 10,000 iterations)
Health Quality Ontario (2018) [31]	Cost-utility analysis	Ontario Ministry of Health and Long-Term Care	5 years (reference case)	1.5% per annum (costs and QALYs^a^)	CAD $	2017	Probabilistic sensitivity analysis Deterministic one-way sensitivity analyses Scenario analyses Discount rate sensitivity analyses
Zanaboni et al (2013) [17]	Cost-utility analysis	Health care system; patient perspective (secondary)	16 months (trial based)	Not reported	Euro	2010	Scenario analysis
Hummel et al (2019) [18]	Cost-effectiveness analysis	Medicare (US payer perspective)	Lifetime (25 years)	3% per annum (costs and outcomes)	US $	2017	Deterministic 1-way sensitivity analysis Scenario analyses
Burri et al (2013) [19]	Cost-consequence analysis	UK NHS^b^	10 years (base case); 5 and 15 years (scenario analyses)	3.5% per annum (costs and outcomes)	Pound sterling	2010	Deterministic univariate sensitivity analysis Scenario analyses (time horizon variation)
Kenny et al (2025) [26]	Cost-effectiveness analysis	UK NHS and PSS^c^	Lifetime	3.5% per annum (costs and outcomes)	Pound sterling	2021-2022	Deterministic sensitivity analysis Probabilistic sensitivity analysis Threshold analyses Scenario analyses
de Graaf et al (2024) [32]	Cost-utility analysis	Health care sector; patient perspective (secondary)	24 months (trial based)	Not reported	Euro	2020	Probabilistic sensitivity analysis (bootstrapping; 10,000 iterations)
Chew et al (2022) [15]	Cost-effectiveness analysis	Canadian public health care system	5 years	1.5% per annum (costs and outcomes)	CAD $	2019	Probabilistic sensitivity analysis Deterministic 1-way sensitivity analysis Scenario analyses
Ricci et al (2017) [20]	Cost-utility analysis	Health care system; patient and caregiver perspectives (secondary)	12 months	Not reported	Euro	Not reported	Not reported
Ahmed et al (2026) [30]	Cost-effectiveness analysis	UK NHS and PSS (England and Wales)	Lifetime (base case: 32.2 years; 386 monthly cycles)	3.5% per annum (costs and benefits)	Pound sterling	2022-2023	Probabilistic sensitivity analysis Scenario analyses Deterministic break-even analysis

a QALY: quality-adjusted life-year.

b NHS: National Health Service.

c PSS: personal social service.

#### Type of Economic Evaluation

The included studies used different forms of economic evaluation. Cost-effectiveness analysis was reported in 4 studies [15,18,26,30]. Cost-utility analysis was reported in 5 studies [17,20,21,31,32]. One study reported a cost-consequence analysis [19].

#### Analytical Perspective

Analytical perspectives also differed across studies. Public or health care system perspectives were commonly adopted [15,19,21,31]. UK-based studies adopted a National Health Service and personal social services perspective [26,30]. Hummel et al [18] used a Medicare payer perspective. Three studies incorporated patient and/or caregiver perspectives as secondary analyses [17,20,32].

#### Time Horizon

Time horizons ranged from short-term trial-based follow-up to lifetime modeling. Trial-based evaluations used follow-up periods of 12 months [20], 16 months [17], and 24 months [32]. A 5-year time horizon was used in 3 studies [15,21,31], whereas 1 study used 10-year horizons [19]. Lifetime time horizons were adopted by 3 studies [18,26,30].

#### Discounting

Discount rates were reported in 7 studies and ranged from 1.5% to 4% for both costs and outcomes [15,18,19,21,26,30,31]. Discount rates were not reported in several studies [17,20,32].

#### Currency and Price Year

Reported currencies reflected the study countries’ locations. A price year was explicitly reported in 8 of the 10 studies [17-19,21,26,30-32], while the remaining studies did not provide a clear price year, limiting direct cross-study comparability.

#### Sensitivity and Uncertainty Analysis

Sensitivity and uncertainty analysis approaches varied across studies. Probabilistic sensitivity analysis was reported in 6 studies [15,21,26,30-32]. Deterministic, one-way, or scenario analyses were also commonly used [15,18,19,26,30,31]. One study used bootstrapping as the basis for probabilistic uncertainty analysis [32], and one study did not report any sensitivity or uncertainty analyses [20].

### Model Structure

Table 5 summarizes the structural characteristics of the model-based evaluations, including modeling approach, model type, cycle length, and health state specification.

**Table 5.** Model structure characteristics of model-based economic evaluations^a^.

Study ID	Study (year)	Modeling approach	Model type	Cycle length	Health states defined
1	Sequeira et al (2020) [21]	Systematic review with meta-analysis of RCTs^b^; de novo cost-utility analysis	Cohort Markov model	1 month	Stable outpatient Cardiovascular hospitalization Death (absorbing state)
2	Health Quality Ontario (2018) [31]	HTA^c^; de novo decision-analytic modeling informed by systematic review, meta-analysis, and administrative data	Two parallel state-transition Markov cohort models: Model 1: ICD^d^/CRT-D^e^ recipients Model 2: pacemaker recipients; 10,000 Monte Carlo simulations	1 month	Model 1 (ICD/CRT-D): Stable HF^f^ (by NYHA^g^ class; transitions between classes permitted) Posthospitalization Death (absorbing state) Model 2 (pacemakers): Stable arrhythmia Posthospitalization (nonstroke) Poststroke Death (absorbing state)
4	Hummel et al (2019) [18]	Patient-level decision-analytic modeling using observational cohort data (PREDICT RM database)	DICE^h^ simulation (individual patient, continuous time event tracking)	Not applicable—DICE simulation uses continuous time event tracking; discrete cycle lengths are not used	No discrete Markov health states defined; model based on individual patient characteristics and time-to-event outcomes (including mortality and rehospitalization)
5	Burri et al (2013) [19]	Decision analytic modeling informed by pooled patient-level data from RCTs	Deterministic cohort Markov model	1 year	Well (stable) Poststroke Postacute decompensated heart failure Death (absorbing state)
6	Kenny et al (2025) [26]	Systematic review and de novo cost-effectiveness analysis (NICE^i^Technology Assessment)	Cohort Markov model	1 month	Alive Dead (absorbing state)
8	Chew et al (2022) [15]	Population-based cohort economic evaluation using administrative health data	Cohort Markov model with parametric survival analysis	1 month	Alive Dead (absorbing state)
10	Ahmed et al (2026) [30]	Regression-based decision-analytic modeling using real-world matched cohort data (TriageHF Plus service evaluation)	Two-state, regression-based, Markov-type model	1 month	Alive Dead (absorbing state); note: NYHA class–informed utility weighting but was not modeled as a discrete transition state

a Study IDs correspond to Table 2. This table presents the 7 model-based economic evaluations.

b RCT: randomized controlled trial.

c HTA: health technology assessment.

d ICD: implantable cardioverter-defibrillator.

e CRT-D: cardiac resynchronisation therapy defibrillator.

f HF: heart failure.

g NYHA: New York Heart Association.

h DICE: discrete individual cost-effectiveness.

i NICE: National Institute for Health and Care Excellence.

#### Modeling Approach and Model Type

Seven studies reported formal model-based economic evaluations (Table 5). Five used state transition or cohort-based Markov models [15,19,21,26,31]. In contrast, one study used a regression-based decision analytic approach operationalized as a 2-state Markov-type model based on matched real-world cohort data [30]. Hummel et al [18] used a patient-level DICE simulation based on observational cohort data rather than a conventional discrete state transition framework.

#### Cycle Length

A 1-month cycle length was reported in 5 models [15,21,26,30,31]. Burri et al [19] used a 1-year cycle length. In contrast, Hummel et al [18] did not apply a conventional cycle-based structure because the DICE simulation used continuous-time event tracking rather than fixed transition cycles.

#### Health State Specification

Health state structures ranged from simple 2-state models to more complex multistate frameworks. Two-state alive-or-dead structures were used in 3 studies [15,26,30]. However, Ahmed et al [30] used NYHA class-informed utility weighting rather than modeling it as a discrete transition state. A multistate structure was used to represent disease progression and clinical events, including stable outpatient status, cardiovascular hospitalization, and death [21]. Additionally, the Health Quality Ontario HTA report described parallel disease-specific models comprising NYHA-based HF progression and posthospitalization states for ICD or CRT-D recipients and stable arrhythmia, posthospitalization, poststroke, and death for pacemaker recipients [31]. One study used stable, poststroke, and postacute decompensated HF and death [19]. Hummel et al [18] did not define discrete Markov health states as the model was structured around individual patient characteristics and time-to-event outcomes, including mortality and rehospitalization.

### Clinical and Economic Outcomes

This section summarizes the clinical and economic outcomes reported across the included studies (Table 6).

**Table 6.** Clinical and economic outcomes reported in included studies.

Study (year)	Clinical outcomes reported	Economic outcomes reported
Sequeira et al (2020) [21]	All-cause mortality Cardiovascular mortality MACE^a^ Composite cardiovascular endpoints	Annual cost per patient Labor costs 5-year cost saving QALYs^b^ ICER^c^ PSA^d^
Health Quality Ontario (2018) [31]	Inappropriate ICD^e^ shocks Clinic visits avoided Time to detection and treatment Major adverse cardiovascular events Arrhythmia burden All-cause mortality Cardiovascular mortality	Total costs Incremental cost QALYs Incremental QALYs ICER; CAD $/QALY Probability of cost-effectiveness Budget impact analysis (5 years)
Zanaboni et al (2013) [17]	ED^f^ and urgent in-office visits HF-related ED or urgent visits Arrhythmia, ICD-related ED, or urgent visits Total health care utilizations Deaths QALYs Utility scores	Annual health care system costs per patient Protocol-defined clinic visit costs ED and urgent visit costs Nonurgent in-office visit costs Scheduled and unscheduled remote follow-up costs Hospitalization costs Diagnostic examination costs Annual patient costs Cost-utility ratio (€/QALY)
Hummel et al (2019) [18]	LYs^g^ Time to death Time to first rehospitalization Number of rehospitalization events	Lifetime costs Costs per patient-year QALYs Incremental cost per life-year gained mental cost per life-year gained ICER; US $/QALY
Burri et al (2013) [19]	All-cause mortality Stroke events ADHF^h^ events SVA^i^ AF^j^ Appropriate and inappropriate ICD shocks Battery replacement events Lead-related complications Scheduled and unscheduled follow-up visits	10-year total costs (discounted and undiscounted) Device costs Patient management costs Cardiovascular event costs
Kenny et al (2025) [26]	Heart failure events All-cause mortality Morbidity and adverse events Patient-reported outcomes Prognostic accuracy of algorithms	Total lifetime costs QALYs Incremental costs Incremental QALYs ICER; £/QALY Cumulative hospitalizations and hospital days Probability of cost-effectiveness Cost breakdown by component
de Graaf et al (2024) [32]	Cardiac-related resource use Hospital admissions Length of hospital stay Outpatient visits Telephone consultations Emergency room visits Ambulance use Patient travel time QALYs	Cardiac-related health care costs Incremental costs Incremental QALYs Commercial headroom analysis Probability of cost-effectiveness
Chew et al (2022) [15]	All-cause mortality Time to first cardiovascular hospitalization Cardiovascular hospitalization rate Hospital length of stay Emergency department visits Outpatient physician visits QALYs Survival	Inpatient admission costs Emergency department visit costs Outpatient physician visit costs Total 5-year costs Incremental cost savings Probability of cost savings
Ricci et al (2017) [20]	In-hospital device follow-up visits Cardiovascular hospitalizations Emergency department visits Outpatient diagnostic tests Outpatient visits QALYs	Cardiovascular hospitalization costs Device-related costs Emergency visit costs Outpatient evaluation and diagnostic test costs Scheduled and unscheduled in-office follow-up costs Total health care system costs Patient travel costs Patient and caregiver productivity loss costs Mean annual patient cost
Ahmed et al (2026) [30]	Unplanned all-cause hospital admissions Emergency department attendances Outpatient cardiology clinic attendances All-cause mortality Heart failure alert events HRQoL^k^ QALYs	Incremental total costs Incremental QALYs ICER; pound sterling/QALY NMB^l^ NHB^m^ Probability of cost-effectiveness Probability of cost-saving Incremental hospitalizations avoided

a MACE: major adverse cardiovascular events.

b QALY: quality-adjusted life-year.

c ICER: incremental cost-effectiveness ratio.

d PSA: probabilistic sensitivity analysis.

e ICD: implantable cardioverter-defibrillator.

f ED: emergency department.

g LY: life-years.

h ADHF: acute decompensated heart failure.

i SVA: supraventricular arrhythmia.

j AF: atrial fibrillation.

k HRQoL: health-related quality of life.

l NMB: net monetary benefit.

m NHB: net health benefit.

#### Clinical Outcomes and Service or Resource Use

Across the included studies, reported outcomes spanned several domains, most commonly mortality or survival, health care use, health-related quality of life, and device- or arrhythmia-related events. Mortality-related outcomes included survival, all-cause and cardiovascular mortality, and time-to-event measures such as time to death [15,18,19,21,30]. Health care use outcomes included hospital admissions, rehospitalization, time to first rehospitalization, length of stay, emergency department visits, outpatient and clinic visits, and other measures of service use [15,17,20,30-32]. Several studies reported health-related quality of life and utility-related outcomes, while a smaller subset reported device- or arrhythmia-related outcomes such as arrhythmia burden, atrial fibrillation, lead-related complications, and battery replacement events [15,17-19,21,26,30-32].

#### Economic Outcomes

Reported economic outcomes included total and incremental costs, incremental cost-effectiveness ratios (ICERs), QALYs, incremental QALYs, cost-utility ratios, probability of cost-effectiveness, and sensitivity or uncertainty analysis outputs such as probabilistic sensitivity analysis. Some studies also reported cost component breakdowns, net monetary benefit, and net health benefit [26,30-32]. Economic end points varied across studies by evaluation framework, outcome measure, and analysis time horizon.

#### Main Economic Findings

Table 7 summarizes the main economic findings, ICER results, and health economic decisions across the included studies.

**Table 7.** Main economic findings, ICER (incremental cost-effectiveness ratio) results, and health economic decisions.

Study (year)	Main economic finding	ICER and/or cost result	Health economic decision
Sequeira et al (2020) [21]	RM^a^ was associated with lower costs and higher QALYs^b^ compared with standard quarterly in-clinic follow-up over 5 years. RM was dominant across the base case.	Dominant (lower cost or higher QALYs)	Dominant
Health Quality Ontario (2018) [31]	ICD^c^/CRT-D^d^: RM was associated with higher costs and higher QALYs; cost-effective at WTP^e^ CAD $50,000/QALY (US $51,697/QALY; 2024); 71% of simulations. Pacemakers: RM was dominant (lower costs and higher QALYs). Budget impact: net saving of CAD $14.18 million over 5 years (US $14.66 million; 2024). Approximately 15% of simulations classified RM as inferior for ICD/CRT-D.	ICD/CRT-D: CAD $23,374/QALY (US $24,167/QALY; 2024). Pacemakers: dominant.	Pacemakers: dominant in the model. ICD/CRT-D: cost-effective at CAD $50,000/QALY, with decision uncertainty .
Zanaboni et al (2013) [17]	RM was associated with lower costs (€888.10 cost saving per patient; US $1586.91; 2024) and higher QALYs (+0.065) compared with conventional follow-up. Dominant result was maintained in the €900 tariff scenario (US $1608.17; 2024). Patient costs were also reduced.	Dominant (lower cost and higher QALYs)	Dominant in the reported cost-utility analysis
Hummel et al (2019) [18]	RPM was associated with increased life-years (*Δ*+0.77 LYs), increased QALYs (*Δ*+0.64), and higher total lifetime costs (*Δ*+ US $6914; US $8655.57; 2024) compared with no RPM	US $10,752/QALY (US $13,460.33/QALY; 2024).	Cost-effective; threshold dependent (favorable at WTP US $50,000/QALY)
Burri et al (2013) [19]	Home monitoring was cost neutral over 10 years compared with conventional in-clinic follow-up, with a minor discounted cost advantage of £33 per patient (US $67.67; 2024). No formal ICER was calculated given the cost-consequence design.	Not applicable (cost-consequence analysis; no ICER calculated)	Cost neutral with minor cost advantage for home monitoring
Kenny et al (2025) [26]	HeartLogic: dominant; incremental QALYs +0.01; reduced hospitalizations and hospital days. TriageHF Plus: dominant; incremental cost −€9048 (−US $15,050.10; 2024 prices); incremental QALYs +0.01; reduced hospitalizations. CorVue: cost-increasing; incremental cost +€37 (+ US $61.54; 2024); incremental QALYs=0. HeartInsight: cost-increasing; incremental cost +€568 (+ US $944.79; 2024); incremental QALYs=0.	HeartLogic: dominant; TriageHF Plus: dominant; CorVue: cost increasing; no QALY gain; HeartInsight: cost increasing; no QALY gain	HeartLogic and TriageHF Plus: modeled as dominant but highly uncertain due to limited comparative evidence and high risk of bias. CorVue and HeartInsight: no robust cost-effectiveness conclusion because comparative outcome evidence was insufficient
de Graaf et al (2024) [32]	RPM^f^ was associated with lower cardiac-related health care costs and shorter patient travel time in both the Netherlands and Germany. Incremental cost: −€1041 (Netherlands; −US $1558.05; 2024) and −€2865 (Germany; −US $4376.45; 2024 prices). Incremental QALYs: −0.003 (Netherlands) and +0.086 (Germany). No formal ICER was reported; cost-effectiveness was dependent on the acquisition cost of the RPM system.	No ICER reported; cost-effectiveness dependent on RPM acquisition cost	No definitive cost-effectiveness conclusion; dependent on RPM acquisition cost and country context
Chew et al (2022) [15]	RM was associated with lower total 5-year costs and similar QALYs compared with in-clinic follow-up. Incremental cost saving: −CAD $12,195 per person over 5 years (−US $12,216.81; 2024). Probability of cost saving exceeded 99% at 5 years. Hospitalization costs were the primary cost driver.	Incremental cost: −CAD $12,195 per person over 5 years (−US $12,216.81; 2024); QALYs were similar.	Cost-saving with equivalent QALYs
Ricci et al (2017) [20]	RM was cost-saving compared with standard care (SC) from health care system, patient, and caregiver perspectives. Annual health care system costs per patient were reduced by 53.87%; patient costs reduced by 66.44%. Primary driver of savings was reduction in cardiovascular hospitalizations (accounting for 81% of total savings). No significant difference in QALYs between groups.	No ICER calculated; no significant QALY difference	Cost-saving without QALY difference
Ahmed et al (2026) [30]	TriageHF Plus was associated with lower total costs and higher QALYs compared with usual care. Incremental cost: −€3989 (−US $6294.61; 2024); incremental QALYs: +0.01. Positive net monetary benefit confirmed cost-effectiveness. Probability of cost-effectiveness and cost-saving both exceeded conventional thresholds.	Dominant (lower costs and higher QALYs)	Dominant in the model-based analysis

a RM: remote monitoring.

b QALY: quality-adjusted life-year.

c ICD: implantable cardioverter-defibrillator.

d CRT-D: cardiac resynchronisation therapy defibrillator.

e WTP: willingness to pay.

f RPM: remote patient monitoring.

Several studies identified remote monitoring as dominant, defined as lower costs with improved health outcomes. Sequeira et al [21] found that remote monitoring was associated with lower costs and higher QALYs than standard quarterly in-clinic follow-up over 5 years. Zanaboni et al [17] similarly reported lower costs and higher QALYs with remote monitoring than with conventional follow-up.

Ahmed et al [30] reported that TriageHF Plus was associated with lower total costs and higher QALYs than usual care in the model-based analysis. Health Quality Ontario [31] also reported dominant findings for pacemaker recipients, whereas for ICD or CRT-D recipients, remote monitoring was associated with higher costs and higher QALYs.

One study reported remote monitoring as cost-effective. Hummel et al [18] found that remote patient monitoring was associated with higher lifetime costs along with gains in life years and QALYs, resulting in an ICER of US $10,752 per QALY.

Three studies reported cost-neutral or cost-saving findings, but neither showed any additional QALY gains or calculated a formal ICER [15,19,20]. Burri et al [19], using a cost-consequence analysis, found home monitoring to be broadly cost neutral over 10 years, with a small, discounted cost advantage. Chew et al [15] reported lower total 5-year costs and similar QALYs compared with in-clinic follow-up, with hospitalization costs identified as the main cost driver. Ricci et al [20] also reported cost savings from the perspectives of the health care system, patients, and caregivers, with no significant difference in QALYs between groups.

de Graaf et al [32] found lower cardiac-related health care costs in both the Netherlands and Germany. Incremental QALYs differed between settings, with a small QALY loss in the Netherlands (−0.003) and a QALY gain in Germany (+0.086). No formal ICER was reported, and the cost-effectiveness conclusion depended on the acquisition cost of the remote monitoring system and the country context [32].

Kenny et al [26] reported algorithm-specific findings. HeartLogic and TriageHF Plus were modeled as dominant, although uncertainty regarding the underlying comparative evidence informing hospitalization and length of stay inputs was reported. CorVue and HeartInsight were associated with higher costs and no QALY gains.

### Reporting and Quality Appraisal

The included studies were evaluated via the CHEQUE assessment, as shown in Figures 2 and 3. As summarized in Table 8, methodology scores ranged from 56.3% to 87.5%, and reporting scores ranged from 70.8% to 83.3%. Overall, reporting scores were generally slightly higher than methodology scores. Full item-level CHEQUE ratings are provided in Table S1 in Multimedia Appendix 1 [15,17-21,26,30-32].

**Figure 2.**
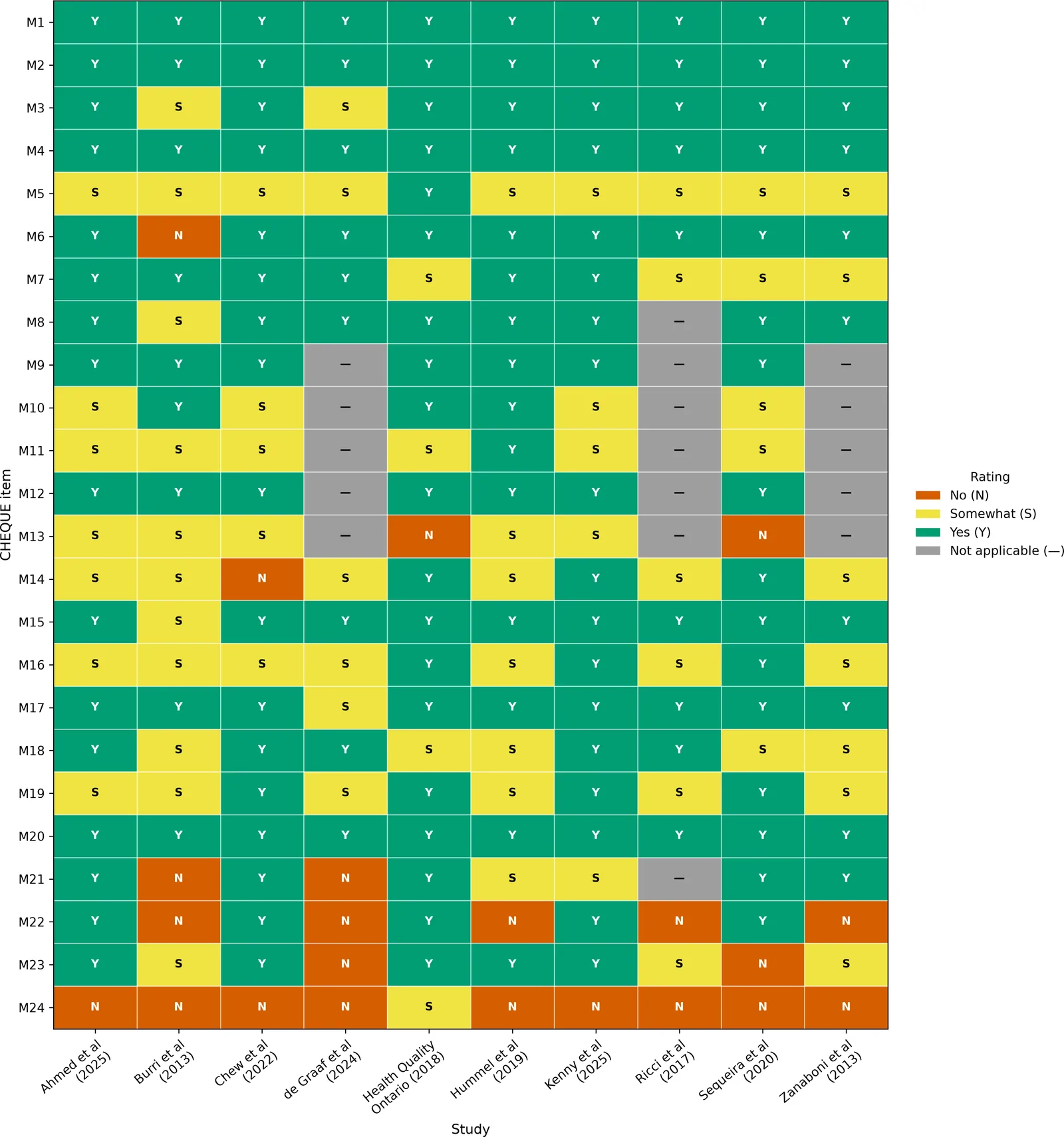
CHEQUE (Criteria for Health Economic Quality Evaluation) methodology item ratings across the included economic evaluations [15,17-21,26,30-32].

**Figure 3.**
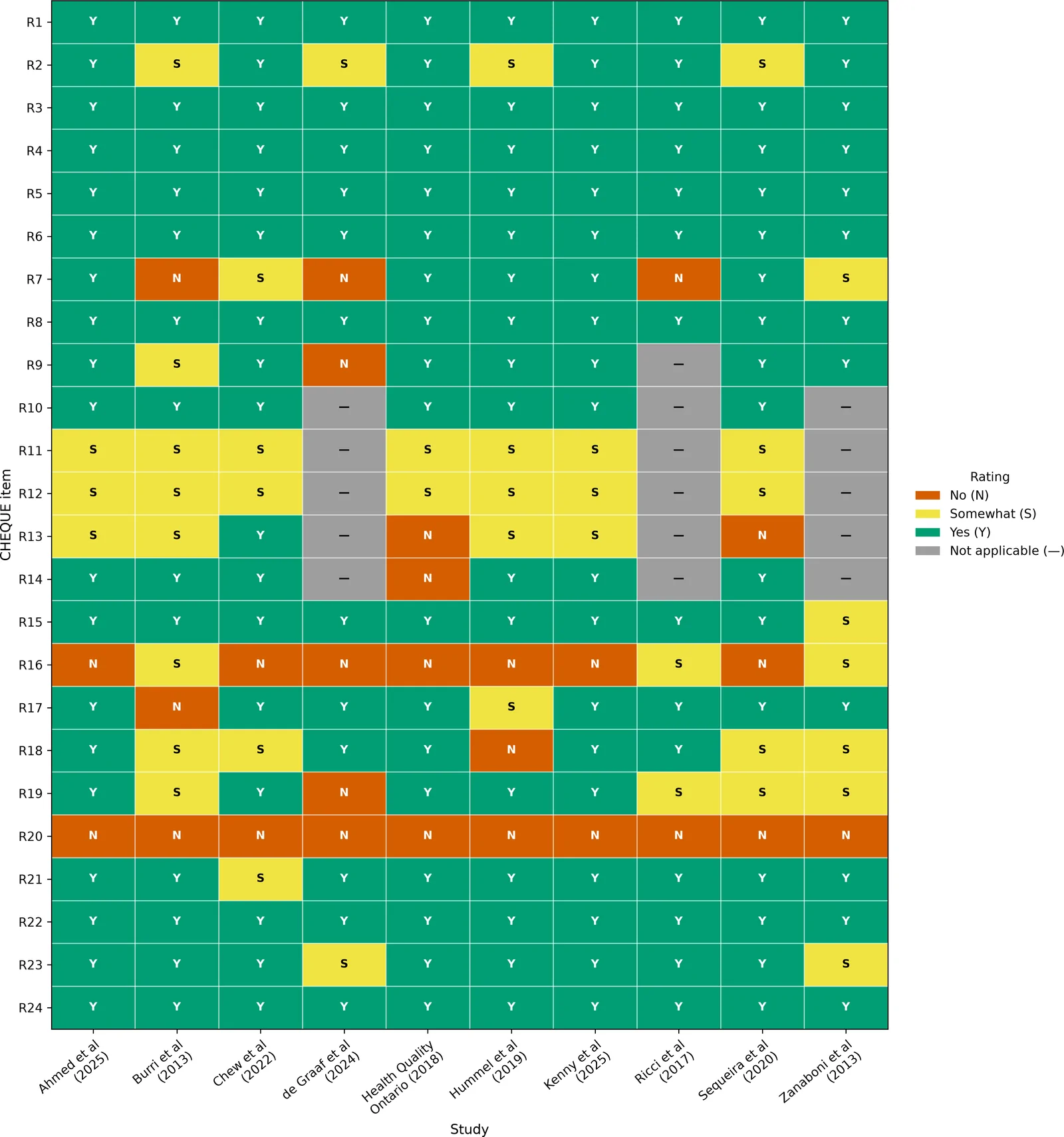
CHEQUE (Criteria for Health Economic Quality Evaluation) reporting item ratings across the included economic evaluations [15,17-21,26,30-32].

**Table 8.** Study-level unweighted CHEQUE (Criteria for Health Economic Quality Evaluation) methods and reporting percentages^a^.

First author (year)	Methods, %	Reporting, %
Sequeira et al (2020) [21]	77.1	75.0
Health Quality Ontario (2018) [31]	85.4	81.3
Zanaboni et al (2013) [17]	65.8	71.1
Hummel et al (2019) [18]	72.9	75.0
Burri et al (2013) [19]	56.3	70.8
Kenny et al (2025) [26]	87.5	83.3
de Graaf et al (2024) [32]	60.5	73.7
Chew et al (2022) [15]	81.3	77.1
Ricci et al (2017) [20]	70.6	77.8
Ahmed et al (2026) [30]	81.3	81.3

a Yes, Somewhat, and No were scored 1, 0.5, and 0, respectively. Methods and reporting percentages were calculated separately as the sum of item scores divided by the number of applicable items, multiplied by 100. Not-applicable items were excluded. All applicable items had equal weight.

The CHEERS-based assessment showed that core elements were generally reported consistently, whereas reporting was more variable for health economic analysis plans, heterogeneity or distributional effects, and patient or stakeholder engagement. Full CHEERS ratings are provided in Table S2 in Multimedia Appendix 1 [15,17-21,26,30-32].

## Discussion

### Principal Findings

This review identified 10 economic evaluations of algorithm-enabled CIED remote monitoring in populations with established HF or considered at risk of HF. The included evaluations differed in population definitions, intervention designs, model structures, time horizons, and reported outcomes, limiting comparability and generalizability. HF status was also defined inconsistently: some evaluations included clearly characterized populations using indicators such as NYHA class or left ventricular ejection fraction, whereas others used less clearly operationalized definitions of populations at risk of HF. Economic findings should therefore be interpreted as context specific rather than directly transferable across populations, algorithms, model structures, and health care settings.

The interventions were conceptually and operationally heterogeneous and should not be treated as a single intervention when assessing cost-effectiveness. They included predefined alert rules and algorithms that combined several measures to classify HF risk [17,19,20,26,30,31]. In some reports, the alert function was described, but the underlying rules were not reported in detail. Monitored parameters ranged from device-related measures to physiological and broader clinical indicators [17,19,26,30-32], while clinical response pathways varied from structured clinician-led review and treatment adjustment to limited reporting of operational processes [17,19,20,30,31]. Economic evaluations reported inconsistently on key implementation and performance features, including alert burden, false-positive alerts, and how alerts were reviewed and acted upon. This limits interpretation of whether favorable economic findings were driven by the algorithm itself, the surrounding clinical pathway, or both.

Economic methods and study contexts varied across the included studies, including patient populations, intervention definitions, evaluation type, analytical perspective, model structure, time horizon, discounting, uncertainty analysis, and clinical input sources [15,17-21,26,30-32]. Favorable findings appeared in both trial-based and model-based evaluations, although results were not consistently cost saving or dominant. In several evaluations, favorable estimates were linked to reported or assumed reductions in hospitalizations, length of stay, or other types of health care use [15,17,18,20,26,30]. QALY gains were often modest; Ahmed et al [30], for example, reported an incremental gain of 0.01 QALYs per patient over the modeled lifetime horizon, while Chew et al [15] reported similar QALYs between groups.

Some evaluations used evidence from the same clinical trial, but they did not use identical economic analyses or reach identical conclusions. Zanaboni et al [17] evaluated the EVOLVO trial. Sequeira et al [21] used EVOLVO utility values alongside ECOST evidence, while Health Quality Ontario [31] used EVOLVO data on unscheduled health care use. The overlap concerns specific clinical inputs. These inputs should not be interpreted as fully independent evidence, but the 3 evaluations were counted and interpreted separately.

The economic results were sensitive to assumptions about baseline hospitalization risk, length of stay, unit costs, clinical response, and the duration of any benefit. This was particularly relevant in long-term models that extended beyond the period of observed clinical data. Comparative evidence on hospitalization and other clinical outcomes was limited for some technologies [26]. For de Graaf et al [32], conclusions also depended on the cost of monitoring and the country setting. These factors restrict the transfer of an estimate from one health care system to another.

Markov-type approaches predominated, ranging from simple two-state alive-or-dead models to more detailed multistate and multiphase frameworks. One study used a patient-level DICE simulation, which tracked mortality and rehospitalization events in continuous time rather than through fixed model cycles [18]. More detailed structures can represent differences in events and their timing, but they require additional data and assumptions. Model structure should therefore be judged against the decision problem and available evidence. Differences in structure also limit direct comparison of costs and QALYs across evaluations.

The included monitoring systems also differed in their function and value mechanism. Where predefined alerts were described, remote monitoring could support earlier review and reduce scheduled in-person visits or other service use [17,19,20,31]. For multiparameter risk systems, economic estimates were particularly sensitive to the assumed or observed effects on hospitalizations and length of stay [26,30]. This suggests that economic value should not be attributed to algorithm-enabled remote monitoring as a single intervention. It depends on system function, alert management, clinical response, evidence strength, implementation cost, and patient population.

Interpretation is further complicated by variation in the populations studied. Included studies covered patients with established HF and those at risk of HF across device groups, including ICDs, CRT-Ds, CRT-Ps, and pacemakers. Because clinical trajectories, monitoring needs, baseline event rates, and downstream costs may differ across these groups, findings should not be assumed to apply equally across all CIED populations. Health Quality Ontario [31], for example, estimated higher costs and higher QALYs for ICD or CRT-D recipients but lower costs and higher QALYs for pacemaker recipients. A result for one device group should not be assumed to apply to all people with HF.

The appraisal findings further limit the strength of a general economic conclusion. Burri et al [19] and de Graaf et al [32] had lower unweighted CHEQUE method percentages. The model by Burri et al [19] predicted cost neutrality under assumptions that did not include a reduction in HF events or mortality. The conclusions by de Graaf et al [32] depended in part on monitoring costs and country context. Kenny et al [26] had a higher method percentage, but comparative outcome evidence for the algorithms remained limited. The favorable cost-effectiveness estimates reported by Kenny et al [26] for HeartLogic and TriageHF Plus should be interpreted in that context. For CorVue and HeartInsight, sufficient comparative outcome evidence was unavailable to produce evidence-based cost-effectiveness estimates. A scenario assuming no clinical benefit produced higher costs without QALY gains [26]. Evaluations using clinical evidence from nonrandomized studies may also be affected by residual confounding. Several evaluations reported manufacturer funding. This warrants careful examination of assumptions and reporting but does not itself establish bias.

### Implications for Policy and Practice

Decision-makers should interpret cost-effectiveness estimates in relation to the monitoring system and the local care pathway. Several evaluations reported favorable findings, while others were cost neutral, mixed, or dependent on costs and assumptions [19,26,31,32]. Local staffing, alert workload, hospitalization costs, and existing monitoring infrastructure may affect whether routine care can achieve a reported benefit. Estimates from one device group or health care setting should therefore not be applied automatically to another. This is consistent with methodological work showing that the value of digital health interventions depends on how they are implemented and integrated into clinical pathways [10,11].

The review also indicates that costs beyond the health care payer perspective may be relevant to decision-making. Some studies reported reductions in patient and caregiver costs, including travel, time, and attendance burden [17,20,32]. A broader analytical perspective that includes patient, caregiver, and societal costs may therefore better capture the value of remote monitoring, particularly where it reduces the need for in-person follow-up [10-12].

### Implications for Future Research

Future evaluations should describe the monitoring intervention and its clinical pathway more clearly. Reports should specify the data used, the conditions that generate alerts, the output shown to clinicians, and the actions taken after review. A commercial algorithm name is less informative than a clear description of what the system does. Consistent reporting of alert frequency, false positives, and staff workload would also help explain economic results across settings.

Greater attention is also needed to patient heterogeneity. Most studies reported average cost-effectiveness results and rarely examined whether costs, outcomes, or economic value differed across clinically relevant subgroups. This gap matters because the value of remote or algorithm-enabled monitoring may vary by device type, HF severity, prior hospitalization, baseline risk, and comorbidity burden. Future evaluations should therefore incorporate subgroup analyses where feasible and examine how structural uncertainty and long-term extrapolation affect conclusions across patient groups.

Models should provide clear reasons for their structural choices and test influential assumptions. Validation and uncertainty analysis should be reported transparently. Real-world data may improve relevance to routine care provided that selection bias and confounding are addressed.

### Strengths and Limitations

This review focused on economic evaluations of algorithm-enabled CIED monitoring in HF-relevant populations. Multiple bibliographic databases and HTA sources were searched. The protocol was registered prospectively. This review describes intervention functions, monitored measures, clinical pathways, economic methods, and findings. It used the CHEQUE checklist for methodological appraisal and the CHEERS 2022 statement to assess completeness of reporting.

Several limitations should also be acknowledged. One reviewer conducted screening and data extraction, with verification rather than full independent duplicate review, which may have increased the risk of missed studies or extraction errors. The CHEQUE and CHEERS assessments were completed by one reviewer. CHEQUE percentages were calculated with equal weights and should be interpreted descriptively. The review was restricted to English-language publications and may therefore have excluded relevant evidence published in other languages.

The evaluations also differed substantially in interventions, populations, settings, perspectives, time horizons, and outcomes. These differences prevented a meaningful pooled economic estimate. Some evaluations used inputs from the same clinical trials, so parts of the underlying evidence were not independent. Incomplete intervention reporting and limited comparative clinical evidence constrained interpretation. No formal certainty of evidence assessment was conducted. These limitations should be considered when judging the overall strength and transferability of the findings.

### Conclusions

Economic evidence on algorithm-enabled CIED remote monitoring in HF-relevant populations appears promising. Most studies reported cost-effective, cost-saving, or dominant findings, with reductions in hospitalization and health care use acting as important drivers in several evaluations. However, the evidence remains heterogeneous and difficult to compare directly across studies. Reported findings were sensitive to differences in intervention design, model structure, analytical assumptions, and clinical input data, which limits certainty and transferability. More robust and standardized evidence is needed before economic conclusions can be applied confidently across different devices, algorithms, and health care settings.

## Supplementary material

10.2196/106939 Multimedia Appendix 1 Detailed search strategies and study-level CHEQUE quality appraisal and CHEERS 2022 reporting assessments.

10.2196/106939 Checklist 1 PRISMA 2020 checklist.

## Abbreviations

CHEERS: Consolidated Health Economic Evaluation Reporting Standards
CHEQUE: Criteria for Health Economic Quality Evaluation
CIED: cardiac implantable electronic device
CRT-D: cardiac resynchronization therapy defibrillator
CRT-P: cardiac resynchronization therapy pacemaker
ECOST: Effectiveness and Cost of ICD Follow-up Schedule With Telecardiology
EVOLVO: Evolution of Management Strategies of Heart Failure Patients With Implantable Defibrillators
HF: heart failure
HTA: health technology assessment
ICD: implantable cardioverter-defibrillator
ICER: incremental cost-effectiveness ratio
NYHA: New York Heart Association
PRISMA: Preferred Reporting Items for Systematic Reviews and Meta-Analyses
PRISMA-P: Preferred Reporting Items for Systematic Reviews and Meta-Analyses Protocols
QALY: quality-adjusted life year
