# Supplementary material for: Economic Evaluations of Algorithm-Enabled Remote Monitoring of Cardiac Implantable Electronic Devices: Systematic Review
Source: J Med Internet Res. 2026 Nov 13;28:e106939. doi: 10.2196/106939 (PMC13766657; doi:10.2196/106939)
Supplement: Supplementary file 2 [file jmir-v28-e106939-s001.docx]

**Appendix (A)**

**Database:**
Embase <1974 to 2025 November 25>

| **#** | **Query** | **Results from 28 Nov 2025** |
| --- | --- | --- |
| 1 | Cardiac Resynchronization Therapy Devices/ | 14,071 |
| 2 | Defibrillators, Implantable/ | 54,089 |
| 3 | Pacemaker, Artificial/ | 34,294 |
| 4 | ("pacemaker*" or "cardiac implantable electronic devices" or "cardiac implant* electric device*" or "implantable cardioverter-defibrillator* " or "implantable cardiac defibrillator" or "Implantable defibrillator*" or "implantable cardioverter-defibrillator*" or "implantable cardioverter-defibrillator " or "cardiac resynchroni?ation therap*" or "cardiac resynchroni?ation therap* " or "resynchroni?ation device*").ti,ab,kf. | 114,263 |
| 5 | 1 or 2 or 3 or 4 | 169,382 |
| 6 | Algorithms/ | 457,634 |
| 7 | Telemedicine/ | 57,767 |
| 8 | Remote Sensing Technology/ | 22,477 |
| 9 | Telemetry/ | 23,554 |
| 10 | ("risk model*" or "predict* model*" or "risk stratification" or "risk predict*" or "risk score*" or "predict* algorithm*" or "algorithm*" or "novel algorithm" or "decision algorithm*" or "decision rule*" or "clinical decision rule*" or classifier* or "scoring system*" or "decision support" or "decision-support telemedicine" or "telehealth" or "tele-health" or "eHealth" or "e-health" or "mHealth" or "m-health" or "digital health" or "virtual care" or "remote consult*" or "video visit*" or "teleconsult*" or "remote sensing" or sensor* or biosensor* or "physiolog* sensor*" or "sensing technolog*" or "physiolog* monitoring" or "device* telemetry" or biotelemetry or "wireless telemetry" or "remote transmission" or "automatic transmission" or "device interrogation").ti,ab,kf. | 1,803,036 |
| 11 | ("remote monitor*" or "remote patient monitor*" or "remote follow-up" or "remote interrogation" or "home monitor*" or "device follow-up" or "remote cardiac monitor*" or "remote device monitor*" or "telemonitor*").ti,ab,kf. | 21,951 |
| 12 | 6 or 7 or 8 or 9 or 10 or 11 | 1,999,803 |
| 13 | Economics/ | 248,302 |
| 14 | Economics, Nursing/ | 37,841 |
| 15 | Economics, Medical/ | 37,841 |
| 16 | Economics, Pharmaceutical/ | 25,824 |
| 17 | exp Economics, Hospital/ | 1,183,478 |
| 18 | Economics, Dental/ | 37,841 |
| 19 | exp "Fees and Charges"/ | 47,469 |
| 20 | exp Budgets/ | 37,716 |
| 21 | Cost-Effectiveness Analysis/ | 217,661 |
| 22 | Cost-Benefit Analysis/ | 102,495 |
| 23 | "Costs and Cost Analysis"/ | 66,964 |
| 24 | "budget*".ti,ab,kf. | 55,509 |
| 25 | exp models, economic/ | 5,317 |
| 26 | markov chains/ | 14,252 |
| 27 | markov.ti,ab,kf. | 44,933 |
| 28 | monte carlo method/ | 60,235 |
| 29 | monte carlo.ti,ab,kf. | 74,071 |
| 30 | exp Decision Theory/ | 1,937 |
| 31 | (decision* adj2 (tree* or analy* or model*)).ti,ab,kf. | 71,593 |
| 32 | ("economic*" or "cost" or "costs" or "costly" or "costing" or "price" or "prices" or "pricing" or "pharmacoeconomic*" or "pharmaco-economic*" or "expenditure" or "expenditures" or "expense" or "expenses" or "financial" or "finance" or "finances" or "financed").ti,kf. | 397,685 |
| 33 | (economic* or cost or costs or costly or costing or price or prices or pricing or pharmacoeconomic* or pharmaco-economic* or expenditure or expenditures or expense or expenses or financial or finance or finances or financed).ab. /freq=2 | 655,259 |
| 34 | (cost* adj2 (effective* or utilit* or benefit* or minimi* or analy* or outcome or outcomes)).ab,kf. | 366,481 |
| 35 | (value adj2 (money or monetary)).ti,ab,kf. | 4,770 |
| 36 | ("quality-adjusted life years" or "Incremental cost-effectiveness ratio" or "Markov").ti,ab,kf. | 65,072 |
| 37 | 13 or 14 or 15 or 16 or 17 or 18 or 19 or 20 or 21 or 22 or 23 or 24 or 25 or 26 or 27 or 28 or 29 or 30 or 31 or 32 or 33 or 34 or 35 or 36 | 2,046,890 |
| 38 | 5 and 12 and 37 | 1,241 |

**Database:**
Ovid MEDLINE(R) <1946 to December Week 2 2025>



| **#** | **Query** | **Results from 16 Dec 2025** |
| --- | --- | --- |
| 1 | Cardiac Resynchronization Therapy Devices/ | 1,718 |
| 2 | Defibrillators, Implantable/ | 20,672 |
| 3 | Pacemaker, Artificial/ | 29,790 |
| 4 | ("pacemaker*" or "cardiac implantable electronic devices" or "cardiac implant* electric device*" or "implantable cardioverter-defibrillator* " or "implantable cardiac defibrillator" or "Implantable defibrillator*" or "implantable cardioverter-defibrillator*" or "implantable cardioverter-defibrillator " or "cardiac resynchroni?ation therap*" or "cardiac resynchroni?ation therap* " or "resynchroni?ation device*").ti,ab,kf. | 60,799 |
| 5 | 1 or 2 or 3 or 4 | 75,922 |
| 6 | Algorithms/ | 340,811 |
| 7 | Telemedicine/ | 46,853 |
| 8 | Remote Sensing Technology/ | 5,027 |
| 9 | Telemetry/ | 10,445 |
| 10 | ("risk model*" or "predict* model*" or "risk stratification" or "risk predict*" or "risk score*" or "predict* algorithm*" or "algorithm*" or "novel algorithm" or "decision algorithm*" or "decision rule*" or "clinical decision rule*" or classifier* or "scoring system*" or "decision support" or "decision-support telemedicine" or "telehealth" or "tele-health" or "eHealth" or "e-health" or "mHealth" or "m-health" or "digital health" or "virtual care" or "remote consult*" or "video visit*" or "teleconsult*" or "remote sensing" or sensor* or biosensor* or "physiolog* sensor*" or "sensing technolog*" or "physiolog* monitoring" or "device* telemetry" or biotelemetry or "wireless telemetry" or "remote transmission" or "automatic transmission" or "device interrogation").ti,ab,kf. | 1,017,543 |
| 11 | ("remote monitor*" or "remote patient monitor*" or "remote follow-up" or "remote interrogation" or "home monitor*" or "device follow-up" or "remote cardiac monitor*" or "remote device monitor*" or "telemonitor*").ti,ab,kf. | 9,525 |
| 12 | 6 or 7 or 8 or 9 or 10 or 11 | 1,214,243 |
| 13 | Economics/ | 27,555 |
| 14 | Economics, Nursing/ | 4,014 |
| 15 | Economics, Medical/ | 9,318 |
| 16 | Economics, Pharmaceutical/ | 3,173 |
| 17 | exp Economics, Hospital/ | 26,345 |
| 18 | Economics, Dental/ | 1,922 |
| 19 | exp "Fees and Charges"/ | 31,780 |
| 20 | exp Budgets/ | 14,434 |
| 21 | Cost-Effectiveness Analysis/ | 1,628 |
| 22 | Cost-Benefit Analysis/ | 99,472 |
| 23 | "Costs and Cost Analysis"/ | 52,409 |
| 24 | "budget*".ti,ab,kf. | 32,063 |
| 25 | exp models, economic/ | 17,062 |
| 26 | exp models, economic/ | 17,062 |
| 27 | markov chains/ | 17,559 |
| 28 | markov.ti,ab,kf. | 25,694 |
| 29 | monte carlo method/ | 34,851 |
| 30 | monte carlo.ti,ab,kf. | 41,635 |
| 31 | exp Decision Theory/ | 14,664 |
| 32 | (decision* adj2 (tree* or analy* or model*)).ti,ab,kf. | 40,598 |
| 33 | ("economic*" or "cost" or "costs" or "costly" or "costing" or "price" or "prices" or "pricing" or "pharmacoeconomic*" or "pharmaco-economic*" or "expenditure" or "expenditures" or "expense" or "expenses" or "financial" or "finance" or "finances" or "financed").ti,kf. | 264,975 |
| 34 | (economic* or cost or costs or costly or costing or price or prices or pricing or pharmacoeconomic* or pharmaco-economic* or expenditure or expenditures or expense or expenses or financial or finance or finances or financed).ab. /freq=2 | 362,817 |
| 35 | (cost* adj2 (effective* or utilit* or benefit* or minimi* or analy* or outcome or outcomes)).ab,kf. | 200,917 |
| 36 | (value adj2 (money or monetary)).ti,ab,kf. | 2,972 |
| 37 | ("quality-adjusted life years" or "Incremental cost-effectiveness ratio" or "Markov").ti,ab,kf. | 36,867 |
| 38 | 13 or 14 or 15 or 16 or 17 or 18 or 19 or 20 or 21 or 22 or 23 or 24 or 25 or 26 or 27 or 28 or 29 or 30 or 31 or 32 or 33 or 34 or 35 or 36 or 37 | 788,289 |
| 39 | 5 and 12 and 38 | 330 |

**Web of Science**

Topic search as recorded in the source notes; execution date not recorded.

TS=((

"pacemaker device*" OR "pacemaker implant*" OR pacemaker* OR

"cardiac implantable electronic devices" OR "cardiac implant* electric device*" OR

"implantable cardioverter-defibrillator*" OR "implantable cardiac defibrillator" OR "implantable defibrillator*" OR defibrillator* OR

"cardiac resynchroni?ation therap*" OR "resynchroni?ation device*" OR "resynchronization therapy"

)

AND

(

algorithm* OR "novel algorithm" OR

"risk model*" OR "risk stratification" OR "risk prediction*" OR "prediction model*" OR "predictive algorithm*" OR

"decision algorithm*" OR "decision rule*" OR "clinical decision rule*" OR classifier* OR "scoring system*" OR

telehealth OR "tele-health" OR telemedicine OR "decision-support telemedicine" OR telemonitor* OR teleconsult* OR

"remote consult*" OR "remote monitor*" OR "remote patient monitor*" OR "remote follow-up" OR

"remote device monitoring" OR "continuous remote monitoring" OR "remote cardiac monitoring" OR

"device telemetry" OR "wireless telemetry" OR sensor* OR biosensor* OR "implant* sensor*" OR "physiolog* sensor*" OR

"remote transmission" OR "automatic transmission" OR "device interrogation" OR "remote interrogation"

)

AND

(

economic* OR cost OR costs OR costly OR costing OR price OR prices OR pricing OR

pharmacoeconomic* OR pharmaco-economic* OR expenditure OR expenditures OR expense OR expenses OR

financial OR finance OR financed OR budget* OR

"cost effective*" OR "cost benefit*" OR "cost utility" OR "cost minim*" OR

"economic model*" OR markov OR "markov model" OR "monte carlo" OR

"decision tree*" OR "decision model*" OR

"quality-adjusted life year*" OR qaly OR "incremental cost-effectiveness ratio"

)

)

**Scopus**

(TITLE-ABS-KEY("cardiac implantable electronic device*" OR CIED* OR

"implantable cardioverter defibrillator*" OR ICD* OR

"cardiac resynchronization therap*" OR CRT OR pacemaker*))

AND

(TITLE-ABS-KEY("algorithm-enabled" OR "risk prediction" OR

"risk stratification" OR "prediction model*" OR

"remote monitoring algorithm*" OR "device algorithm*"))

AND

(TITLE-ABS-KEY("cost-effectiveness" OR "cost utility" OR

"economic evaluation" OR "cost analysis" OR "health economics"))

**Cochrane Library**

*Advanced search (Title, Abstract, Keyword)*

(defibrillator* OR "cardiac resynchronization therapy" OR "cardiac resynchronisation therapy")

AND

("remote monitoring" OR "remote follow-up" OR telemonitor* OR "home monitoring")

AND

(cost* OR economic* OR QALY*)

**Supplementary source search**

**NICE**

("cardiac implantable electronic device*" OR "implantable cardioverter-defibrillator*" OR "cardiac resynchroni?ation therap*" OR defibrillator*)

AND

(telemonitor* OR "remote monitor*" OR telemetry OR "device interrogation" OR "remote transmission" OR algorithm* OR "risk stratification" OR "risk model*" OR "prediction model*")

AND

(economic* OR cost OR costs OR "cost-effect*" OR "cost benefit*" OR "cost utility" OR qaly OR icer OR markov OR "decision tree*")

**INAHTA International HTA Database**

"cardiac implantable electronic device" OR "cardiac resynchronization therapy" OR "cardiac resynchronisation therapy"

**CADTH / CDA-AMC**

cardiac implantable electronic device

implantable cardioverter defibrillator

cardiac resynchronization

**NIHR Journals Library**

"remote monitoring" defibrillator

"cardiac implantable electronic device"

"cardiac resynchronisation therapy"

**NHS Economic Evaluation Database (NHS EED)**

( "cardiac resynchronization therapy" OR "cardiac resynchronisation therapy")

AND

("remote monitoring" OR telemonitor* OR "remote follow-up" OR telemetry)

**Supplementary Table S1. CHEQUE, Criteria for Health Economic Quality Evaluation**

| **Domain** | **Item** | **Type** | **Sequeira et al.**  **(2020)** | **Health Quality**  **Ontario (2018)** | **Zanaboni et al.**  **(2013)** | **Hummel et al.**  **(2019)** | **Burri et al.**  **(2013)** | **Kenny et al.**  **(2025)** | **Gimon de Graaf**  **et al. (2024)** | **Chew et al.**  **(2022)** | **Ricci et al.**  **(2017)** | **Ahmed et al.**  **(2025)** |
| --- | --- | --- | --- | --- | --- | --- | --- | --- | --- | --- | --- | --- |
| Decision Problem and Scope | M1 | Methods | Yes | Yes | Yes | Yes | Yes | Yes | Yes | Yes | Yes | Yes |
| Decision Problem and Scope | M2 | Methods | Yes | Yes | Yes | Yes | Yes | Yes | Yes | Yes | Yes | Yes |
| Decision Problem and Scope | R1 | Reporting | Yes | Yes | Yes | Yes | Yes | Yes | Yes | Yes | Yes | Yes |
| Intervention and Comparator(s) | M3 | Methods | Yes | Yes | Yes | Somewhat | Somewhat | Yes | Yes | Yes | Yes | Somewhat |
| Intervention and Comparator(s) | R2 | Reporting | Somewhat | Yes | Yes | Somewhat | Yes | Somewhat | Yes | Somewhat | Yes | Yes |
| Intervention and Comparator(s) | R3 | Reporting | Yes | Yes | Yes | Yes | Yes | Yes | Yes | Yes | Yes | Yes |
| Perspective | M4 | Methods | Yes | Yes | Yes | Yes | Yes | Yes | Somewhat | Yes | Yes | Yes |
| Perspective | R4 | Reporting | Yes | Yes | Yes | Yes | Yes | Yes | Yes | Yes | Yes | Yes |
| Population | M5 | Methods | Somewhat | Somewhat | Somewhat | Somewhat | Somewhat | Somewhat | Somewhat | Somewhat | Somewhat | Somewhat |
| Population | R5 | Reporting | Yes | Yes | Yes | Yes | Yes | Yes | Yes | Yes | Yes | Yes |
| Outcome Measures | M6 | Methods | Yes | Yes | Yes | Yes | No | Yes | Yes | Yes | Yes | Yes |
| Outcome Measures | R6 | Reporting | Yes | Yes | Yes | Yes | Yes | Yes | Yes | Yes | Yes | Yes |
| Outcome Measures | R7 | Reporting | Yes | Yes | Somewhat | Yes | No | Yes | No | Somewhat | No | Yes |
| Time Horizon | M7 | Methods | Somewhat | Somewhat | Somewhat | Yes | Yes | Yes | Somewhat | Yes | Somewhat | Yes |
| Time Horizon | R8 | Reporting | Yes | Yes | Yes | Yes | Yes | Yes | Yes | Yes | Yes | Yes |
| Discounting | M8 | Methods | Yes | Yes | No | Yes | Somewhat | Yes | No | Yes | N/A | Yes |
| Discounting | R9 | Reporting | Yes | Yes | No | Yes | Yes | Yes | No | Yes | N/A | Yes |
| Modeling | M9 | Methods | Yes | Yes | N/A | Yes | Yes | Yes | N/A | Yes | N/A | Yes |
| Modeling | M10 | Methods | Somewhat | Yes | N/A | Yes | Yes | Somewhat | N/A | Somewhat | N/A | Yes |
| Modeling | M11 | Methods | Somewhat | Somewhat | N/A | Somewhat | Somewhat | Somewhat | N/A | Somewhat | N/A | Somewhat |
| Modeling | M12 | Methods | Yes | Yes | N/A | Yes | Somewhat | Yes | N/A | Yes | N/A | Yes |
| Modeling | M13 | Methods | No | No | N/A | Somewhat | Somewhat | Somewhat | N/A | Somewhat | N/A | Somewhat |
| Modeling | R10 | Reporting | Yes | Yes | N/A | Yes | Yes | Yes | N/A | Yes | N/A | Yes |
| Modeling | R11 | Reporting | Somewhat | Yes | N/A | Somewhat | Somewhat | Somewhat | N/A | Somewhat | N/A | Somewhat |
| Modeling | R12 | Reporting | Somewhat | Somewhat | N/A | Somewhat | Somewhat | Somewhat | N/A | Somewhat | N/A | Somewhat |
| Modeling | R13 | Reporting | No | No | N/A | Somewhat | Somewhat | Somewhat | N/A | Yes | N/A | Somewhat |
| Modeling | R14 | Reporting | Yes | No | N/A | Yes | Yes | Yes | N/A | Yes | N/A | Yes |
| Data Inputs and Evidence Synthesis | M14 | Methods | Yes | Yes | Somewhat | Somewhat | Somewhat | Yes | Somewhat | Somewhat | No | Somewhat |
| Data Inputs and Evidence Synthesis | M15 | Methods | Yes | Yes | Yes | Yes | Somewhat | Yes | Yes | Yes | Yes | Yes |
| Data Inputs and Evidence Synthesis | M16 | Methods | Yes | Yes | Somewhat | Somewhat | Somewhat | Yes | Somewhat | Somewhat | Somewhat | Somewhat |
| Data Inputs and Evidence Synthesis | R15 | Reporting | Yes | Yes | Somewhat | Yes | Yes | Yes | Yes | Yes | Yes | Yes |
| Consequences | M17 | Methods | Yes | Yes | Yes | Yes | Yes | Yes | Somewhat | Yes | Yes | Yes |
| Consequences | R16 | Reporting | No | No | Somewhat | No | Somewhat | No | No | No | Somewhat | No |
| Utilities (Preference Measures) | M18 | Methods | Somewhat | Somewhat | Somewhat | Somewhat | No | Yes | Yes | Yes | Yes | Yes |
| Utilities (Preference Measures) | R17 | Reporting | Yes | Yes | Yes | Somewhat | No | Yes | Yes | Yes | Yes | Yes |
| Costs and Resource Use | M19 | Methods | Yes | Yes | Somewhat | Somewhat | Somewhat | Yes | Somewhat | Somewhat | Yes | Somewhat |
| Costs and Resource Use | R18 | Reporting | Somewhat | Yes | Somewhat | No | Somewhat | Yes | Yes | Somewhat | Somewhat | Somewhat |
| Analysis | M20 | Methods | Yes | Yes | Yes | Yes | Yes | Yes | Yes | Yes | Yes | Yes |
| Analysis | M21 | Methods | Yes | Yes | Yes | Somewhat | No | Somewhat | No | Yes | N/A | Yes |
| Analysis | M22 | Methods | Yes | Yes | No | No | No | Yes | Yes | Yes | No | Yes |
| Analysis | M23 | Methods | No | Yes | Somewhat | Yes | Somewhat | Yes | No | Yes | Somewhat | Yes |
| Analysis | R19 | Reporting | Somewhat | Yes | Somewhat | Yes | Somewhat | Yes | Somewhat | Yes | Somewhat | Yes |
| Equity Considerations | M24 | Methods | No | Somewhat | No | No | No | Somewhat | No | No | No | No |
| Equity Considerations | R20 | Reporting | No | No | No | No | No | No | No | No | No | No |
| Transparency and Reporting | R21 | Reporting | Yes | Yes | Yes | Yes | Yes | Yes | Yes | Somewhat | Yes | Yes |
| Transparency and Reporting | R22 | Reporting | Yes | Yes | Yes | Yes | Yes | Yes | Yes | Yes | Yes | Yes |
| Transparency and Reporting | R23 | Reporting | Somewhat | Yes | Somewhat | Somewhat | Somewhat | Yes | Somewhat | Somewhat | Somewhat | Somewhat |
| Transparency and Reporting | R24 | Reporting | Yes | Yes | Somewhat | Yes | Somewhat | Yes | Yes | Yes | Yes | Yes |

**Supplementary Table S2.** CHEERS 2022 Reporting Assessment Across Included Studies

| **Item** | **CHEERS 2022 Reporting Item** | **Sequeira et al.**  **(2020)** | **Health Quality**  **Ontario (2018)** | **Zanaboni et al.**  **(2013)** | **Hummel et al.**  **(2019)** | **Burri et al.**  **(2013)** | **Kenny et al.**  **(2025)** | **Gimon de Graaf**  **et al. (2024)** | **Chew et al.**  **(2022)** | **Ricci et al.**  **(2017)** | **Ahmed et al.**  **(2025)** |
| --- | --- | --- | --- | --- | --- | --- | --- | --- | --- | --- | --- |
| **1** | Title | Yes | Yes | Yes | Yes | Yes | Yes | Yes | Yes | Yes | Yes |
| **2** | Abstract | Yes | Yes | Yes | Yes | Yes | Yes | Yes | Yes | Yes | Yes |
| **3** | Background and objectives | Yes | Yes | Yes | Yes | Yes | Yes | Yes | Yes | Yes | Yes |
| **4** | Health economic analysis plan | No | No | No | No | No | No | No | No | No | No |
| **5** | Study population | Yes | Yes | Yes | Yes | Yes | Yes | Yes | Yes | Yes | Yes |
| **6** | Setting and location | Yes | Yes | Yes | Yes | Yes | Yes | Yes | Yes | Yes | Yes |
| **7** | Comparators | Yes | Yes | Yes | Somewhat | Yes | Yes | Yes | Yes | Yes | Yes |
| **8** | Perspective | Yes | Yes | Yes | Yes | Yes | Yes | Yes | Yes | Yes | Yes |
| **9** | Time horizon | Yes | Yes | Yes | Yes | Yes | Yes | Yes | Yes | Yes | Yes |
| **10** | Discount rate | Yes | Yes | No | Yes | Yes | Yes | No | Yes | No | Yes |
| **11** | Selection of outcomes | Yes | Yes | Yes | Yes | Yes | Yes | Yes | Yes | Yes | Yes |
| **12** | Measurement of outcomes | Yes | Yes | Yes | Yes | Yes | Yes | Yes | Yes | Yes | Yes |
| **13** | Valuation of outcomes | Yes | Yes | Yes | Yes | N/A | Yes | Yes | Yes | Yes | Yes |
| **14** | Measurement and valuation of resources and costs | Yes | Yes | Yes | Yes | Yes | Yes | Yes | Yes | Yes | Yes |
| **15** | Currency, price date, and conversion | Somewhat | Yes | Yes | Yes | Somewhat | Yes | Yes | Yes | Somewhat | Yes |
| **16** | Rationale and description of model | Yes | Somewhat | N/A | Yes | Yes | Yes | N/A | Yes | Yes | Yes |
| **17** | Analytics and assumptions | Yes | Yes | Yes | Yes | Yes | Yes | Yes | Yes | No | Yes |
| **18** | Characterising heterogeneity | No | No | No | No | Yes | Somewhat | No | Somewhat | No | Yes |
| **19** | Characterising distributional effects | No | No | No | No | No | No | No | No | No | No |
| **20** | Characterising uncertainty | Yes | Yes | Yes | Somewhat | Yes | Yes | Yes | Yes | No | Yes |
| **21** | Approach to engagement with patients and others affected by the study | No | Yes | No | No | No | No | No | No | No | Yes |
| **22** | Study parameters | Yes | Yes | Yes | Yes | Yes | Yes | Yes | Yes | Yes | Yes |
| **23** | Summary of main results | Yes | Yes | Yes | Yes | Yes | Yes | Yes | Yes | Yes | Yes |
| **24** | Effect of uncertainty | Yes | Yes | Yes | No | Yes | Yes | Yes | Yes | No | Yes |
| **25** | Effect of engagement with patients and others affected by the study | No | No | No | No | No | N/A | No | No | No | No |
| **26** | Study findings, limitations, generalisability, and current knowledge | Yes | Yes | Yes | Yes | Yes | Yes | Yes | Yes | Yes | Yes |
| **27** | Source of funding | Yes | Yes | Yes | Yes | Yes | Yes | Yes | Yes | Yes | Yes |
| **28** | Conflicts of interest | Yes | No | Yes | Yes | Yes | No | Yes | Yes | Yes | Yes |
